# Impact of Parental Beliefs on Child Developmental Outcomes: A Quasi-Experiment in Rural China

**DOI:** 10.3390/ijerph19127240

**Published:** 2022-06-13

**Authors:** Lei Wang, Conghong Yang, Dingjing Jiang, Siqi Zhang, Qi Jiang, Scott Rozelle

**Affiliations:** 1 International Business School, Shaanxi Normal University, Xi’an 710119, China; wangleiml@163.com (L.W.); yangconghongy@163.com (C.Y.); jadingjing@163.com (D.J.); 2 Stanford Center on China’s Economy and Institutions, Stanford University, Stanford, CA 94305, USA; rozelle@stanford.edu; 3 School of Public Health, University of California, Berkeley, CA 94720, USA; qi_jiang@berkeley.edu

**Keywords:** parental beliefs, parenting practices, child development, quasi-experiment, rural China

## Abstract

This paper examines the impact of parental beliefs on child development outcomes (for both cognitive and social–emotional skills) based on a three-wave longitudinal survey in rural China. The survey waves were conducted when the sample children were 18–30 months, 22–36 months, and 49–65 months, respectively. A total of 815 children and their primary caregivers who participated in all three wave surveys were enrolled in this study. Using difference-in-differences and propensity score matching approaches, the results indicate that strengthened parental beliefs have a positive and significant impact on child social–emotional development. Specifically, between the periods of the Wave 1 survey (when children were 18–30 months old) and the Wave 3 survey (when children were 49–65 months old), and between the Wave 2 survey (when children were 22–36 months old) and the Wave 3 survey, strengthened parental beliefs were causally associated with more favorable child social–emotional scores by 0.44 SD (*p* < 0.01) and 0.49 SD (*p* < 0.01), respectively. No significant impact, however, was found between the period of the Wave 1 survey and the Wave 2 survey. In contrast, weakened parental beliefs had a negative and significant impact on child social–emotional development. Specifically, weakened parental beliefs were causally associated with worse child social–emotional abilities by 0.35 SD (*p* < 0.01), 0.30 SD (*p* < 0.01), and 0.22 (*p* < 0.05) for the time period of the Wave 1 to Wave 2, Wave 1 to Wave 3, and Wave 2 to Wave 3, respectively. No significant impact of parental beliefs, however, was found on child cognitive development. In addition, the findings of the mediation analysis show that only a marginal impact of parental beliefs on child social–emotional development can be indirectly explained by parental beliefs through parenting practices. This study calls on policy makers to improve parental beliefs and parenting practices in the hope that it will lead to better child development in rural China.

## 1. Introduction

Research has shown that there are high rates of cognitive and non-cognitive developmental delays in children under the age 5 in rural China [1,2,3,4]. According to a systematic review by Emmers et al. (2021), nearly 45% and 36% children under age 5 have delays in cognitive and social–emotional skills, respectively [5]. Due to high brain plasticity in the first years of life, the developmental outcomes at this age are critical to the development of more complex cognitive and social–emotional skills in later childhood and even to the levels of educational attainment and employment in adulthood [6,7,8,9]. Since China’s young rural population is one of the keys to human capital in the future labor force, high rates of developmental delays in the rural children of China will almost surely hinder China’s economy in the future [10,11].

There is mounting evidence that the adoption of effective parenting practices by caregivers in the early years of a child’s life can improve their developmental outcomes and help children to achieve their developmental potential [6,12,13,14,15,16,17]. According to these studies, children who come from either developed or less-developed countries have better development (higher levels of cognitive and social–emotional abilities) if their parents interact with them more frequently or engage them in more reading, playing or other effective parenting activities. Randomized controlled trials providing parenting training to improve caregiver interactions with their children, both in developed and developing countries, have proven that such interventions increase interactions between caregivers and their children under the age of 5 and can lead to higher levels of cognitive and social–emotional development [18,19,20].

Unfortunately, studies have shown that parenting practices are poor in rural China when children are under the age of 5 [1,4,5,21,22]. According to the research, only 23% of caregivers read books to their children, and only 25% of caregivers tell stories to their children [5]. Studies have shown that low levels of interaction between caregivers and their children may be one cause of the high rates of developmental delays seen in young children in rural China [1,4].

While there are different sources of child developmental delays, previous studies have revealed that parental beliefs can affect early childhood development [23,24,25,26]. A U.S. study demonstrated that parental beliefs were positively associated with preschool-aged children’s developmental outcomes, including academic achievement and socio-emotional competencies; specifically, stronger parental beliefs were associated with higher math and reading achievement, as well as improved social–emotional skills [26]. In Ethiopia, a developing country, a study (*N* = 102) found a significant and positive association between parental beliefs and child executive function skills [25].

Many interventions, aimed at changing parenting practices, have shown that one of the factors that could influence the effect of the intervention is parental beliefs [6,27,28,29]. Caregivers who believe in the importance of parenting practices to child development improve their parenting practices more, compared to caregivers who do not have any strong belief in the impact of stimulating interactions between caregiver and child on early childhood development [27]. In other words, research shows that parental beliefs have an indirect impact on child development through parenting practices. For example, one study conducted in the U.S. found that parental beliefs can predict the level of parental investment in their children when the children are under age 5 [30]. A study in Colombia showed that parental beliefs about the process of child development were a key driver of parent–child interactions when the sample children are aged 22–42 months [27]. A study in Russia showed that parental beliefs were linked to child developmental outcomes through parenting practices; specifically, parenting practices for math learning mediated the association between parental beliefs and the numeric skills of their preschool aged children [31].

Even though parental beliefs have been proven to have an impact on child development directly or indirectly, to our knowledge, the impact of parental beliefs on the cognitive and social–emotional development of children under the age of 5 in rural China has not been examined. Additionally, to the best of our knowledge, only one study has investigated the associations between parental beliefs and child development in rural China [32]. However, to date no causal analysis has been conducted.

The present study aims to examine the impact of parental beliefs on child cognitive and social–emotional developmental outcomes in rural China. To achieve this goal, the present study has four specific objectives. First, child cognitive and social–emotional development outcomes were assessed during the research team’s survey using different measurements based on the age ranges of the sample children. Second, parenting practices and parental beliefs were measured by interviewing caregivers. Third, the impact of parental beliefs on child developmental outcomes was examined using a quasi-experimental approach. Finally, the mediation effects of parental beliefs on child development through parenting practices were also investigated. To meet these objectives, this study conducted a three-wave longitudinal cohort survey in rural China. The data were collected when the age of the sample children was between one of three different age ranges (18–30 months old, 22–36 months old, and 49–65 months old). The study included 815 caregiver–child pairs who were tracked in all three waves. Difference-in-differences (DID) and propensity score matching (PSM) methods were used to infer the causal effects of parental beliefs on child cognitive and social–emotional development during the different age ranges. A mediation model was used to examine if parental beliefs affected child development through parenting practices.

The results of this study demonstrate that strengthened parental beliefs have a significant positive impact on child social–emotional development. Specifically, between the periods of the Wave 1 survey (when children were 18–30 months old) and the Wave 3 survey (when children were 49–65 months old), and between the Wave 2 survey (when children were 22–36 months old) and the Wave 3 survey, strengthened parental beliefs were causally associated with more favorable child social–emotional scores by 0.44 SD (*p* < 0.01) and 0.49 SD (*p* < 0.01), respectively. No significant impact, however, was found between the period of the Wave 1 survey and the Wave 2 survey. In the case of cognitive development, no significant impact of parental beliefs was found in any of the studied periods. In addition, the results show that weakened parental beliefs negatively and significantly affected child social–emotional development during all three time periods (the periods of the Wave 1 survey and the Wave 2 survey; the Wave 1 survey and the Wave 3 survey; and the Wave 2 survey and the Wave 3 survey). No significant impacts of weakened parental beliefs were found on child cognitive development between any of the survey periods. In performing the mediation analysis, negative and significant mediating effects of weakened parental beliefs on child social–emotional development through parenting practices were detected between the period of the Wave 1 survey and the Wave 2 survey. However, no such mediating effects were found in other cases.

This study makes two contributions to the literature. First, this is the first longitudinal study to examine the impact of parental beliefs on child developmental outcomes in rural China, and also to examine the dynamic paths of parental beliefs and child developmental outcomes over a relatively long time period (spanning three time periods). Second, using a quasi-experimental method, we were able to identify causal-based effects of parental beliefs on parenting practices and on the developmental outcomes of children in rural (or underdeveloped) regions.

The remainder of this paper is as follows. Section 2 describes the methods that we used. Section 3 presents the results. Section 4 discusses the findings. Section 5 concludes.

## 2. Materials and Methods

### 2.1. Participants

The data presented in this paper are drawn from a three-wave longitudinal study conducted in 11 counties in western rural China. Participants included 815 children and their primary caregivers, who were tracked in all three waves (Wave 1 in October 2014 when children were 18–30 months; Wave 2 on April 2015 when children were 22–36 months; and Wave 3 in June 2017 when children were 49–65 months). In the last wave of the survey, children were 55.81 months on average, 51% of the children were boys, only 4% of the children were born prematurely and about 5% had low birth weight. For household characteristics, the mother was the primary caregiver for 62% of the children. The average age of caregivers was 34.77 years old. Around 34% of the caregivers had not completed 9 years of schooling. 74% of the mothers had completed 9 years of schooling, and 83% of the fathers had completed 9 years of schooling.

### 2.2. Sample Selection

Sample selection for this study was conducted in 2013 and followed a multistage cluster sampling design. First, all townships in 11 counties were included, excluding the township in each county that housed the county seat and those townships that did not have any villages with a population of at least 800. These exclusion criteria were chosen to ensure that the sample would be largely rural and also increase the likelihood that sampled villages had a sufficient number of children aged 6–12 months. Following these criteria, 174 townships were included in this study. Next, in each of the sample townships, two villages were randomly selected. Finally, a list of all registered births, provided by local officials in each sample village, was obtained, and all children aged 6–12 months in the sample villages were included in the study.

Three follow-up surveys (October 2014, when the sample children were 18–30 months old; April 2015, when the sample children were 22–36 months old; and June 2017, when the sample children were 49–65 months old) were completed after the initial survey conducted in 2013. In this study, we used data from the three follow-up surveys, as parental beliefs data were not collected in the initial survey in 2013. In conducting the analysis, the final sample included 815 children and their families who participated in all three follow-up surveys.

### 2.3. Measures

#### 2.3.1. Child Cognitive Development

In each of the three waves, child cognitive development was assessed using different measurements based on their age groups. In Wave 1 (when children were 18–30 months), all children were assessed for their cognitive development using the Bayley Scale of Infant Development (BSID). A standard activity kit was used to directly test different skills of children and enumerators scored children on a standardized form based on their performance on the tested activities. The BSID produces a mental development index (MDI) that is a measure of memory, habitation, problem solving, early number concepts, generalization, classification, vocalizations, and language. The test was formally adapted to the Chinese language and environment in 1993 and scaled according to an urban Chinese sample. In the Chinese version of the BSID, the MDI has an inter-rater reliability of 0.99, a test–retest reliability rate of 0.82, and a parallel forms reliability of 0.85 [33].

In Wave 2 (when children were 22–36 months), children who were under 30 months were assessed using BSID, while the Griffith Mental Development Scale (GMDS-ER 2-8) was used to measure cognitive development for children between 30 and 36 months. The GMDS-ER comprises six subscales: locomotor, personal-social, language (receptive and expressive), hand and eye coordination, performance, and practical reasoning. The Chinese version of GMDS test has a reliability rate of 0.95, and a test–retest reliability rate of 0.95 [34].

In Wave 3 (when children were 49–65 months), all children were assessed using the Chinese version of the Wechsler Preschool and Primary Scale of Intelligence-Fourth Edition (WPPSI-IV). The WPPSI-IV produces a Full-Scale Intelligence Quotient (FSIQ), which is a composite score that provides a summary of cognitive ability across a diverse set of domains. The Chinese version of the WPPSI-IV was adapted in 2010 and scaled according to a Chinese sample from urban and rural areas [35], and has since been applied in research across China [2,36]. The Chinese version of the WPPSI-IV test has a reliability coefficient of 0.96 for the FSIQ [35].

All response scores of each measurement were summed to create raw scores of MDI, GMDS-ER, and FSIQ, respectively. For analysis, in wave 1 and wave 3, since raw scores are increasing in age, we computed age-adjusted z-scores using age-conditional means and standard deviations estimated by non-parametric regression. In Wave 2, we first standardized the scores of the BSID and GMDS-ER separately and then pooled the standardized scores as child cognitive developmental outcomes for the analysis of Wave 2.

#### 2.3.2. Child Social–Emotional Development

In each of the three waves, the Ages and Stages Questionnaire: Social Emotional (ASQ:SE) was used to assess child social–emotional development by interviewing the primary caregiver of each child. The primary caregiver was asked to indicate whether the child exhibited a series of behaviors and was asked to characterize the frequency by the following: “most of the time”, “sometimes”, or “never”. Depending on the desirability of the behavior, the answers were scored 0, 5, or 10 points. The different types of behavior included the ability to calm down, accept directions, demonstrate feelings for others (empathy), communicate feelings, initiate social responses to parents and others, and respond without guidance (move to independence). For analysis, the raw scores of ASQ:SE were standardized separately for each wave. The higher the score, the lower the level of the child social–emotional development.

#### 2.3.3. Parental Beliefs

In each of the three waves, the parental beliefs of the primary caregivers of each child were evaluated by asking the primary caregivers sets of questions (12 items in total), such as “I think it is fun to play games with my child”; “I believe that it is important to play with my child”; “I know how to play with my child”; “I believe that it is important to read books or tell stories to my child”; “I know how to read storybooks to my child”, etc. Primary caregivers used a 5-point scale (1 for “completely incorrect” to 5 for “completely correct”) to score each item. For analysis, the scores of parental beliefs were factor analyzed and standardized separately for each wave [7,37,38].

#### 2.3.4. Parenting Practices

In each of the three waves, a questionnaire was administered to the primary caregivers to assess their parenting practices. The questionnaire included four questions that have been shown to be indicators of parenting practices closely associated with child development [39,40,41]. The primary caregiver of each child was asked if they engaged with their child in any of the following four activities on the day before we delivered the survey to each household: “Did you tell stories to your child yesterday?”; “Did you read books to your child yesterday?”; “Did you sing songs to your child yesterday?”; and “Did you play interactively with your child yesterday?” For analysis, the scores of parenting practices were factor analyzed and standardized separately for each wave.

### 2.4. Statistical Analysis

To estimate the impact of parental beliefs on child developmental outcomes, we adopted a difference-in-differences (DID) method [42,43]. To create the treatment and comparison groups, we first defined “low beliefs” as the parental beliefs score below the mean, and “high beliefs” as the parental beliefs score above the mean. All children were divided into two treatment groups and two comparison groups when comparing the changes in parental beliefs between two waves (Wave 1 and Wave 2; Wave 1 and Wave 3; and Wave 2 and Wave 3). The two sets of treatment and comparison groups were: (a) treatment group 1, consisting of primary caregivers who had low beliefs at the baseline wave and high beliefs at the endline wave (strengthened parental beliefs), and comparison group 1, consisting of primary caregivers who had low beliefs at both waves; and (b) treatment group 2, consisting of primary caregivers who had high beliefs at the baseline wave and low beliefs at the endline wave (weakened parental beliefs), and comparison group 2, consisting of primary caregivers who had high beliefs at both waves.

As shown in Table 1 and Table 2, there is no significant difference between the treatment group and the comparison group when comparing the child characteristics in all three time periods. However, there are significant differences between the treatment group and the comparison group when comparing household characteristics. This finding suggests the importance of controlling for those characteristics when carrying out DID analysis.

We then employed a DID approach to compare the child developmental outcomes during the two different waves between the two sets of treatment groups and comparison groups. This comparison produced a DID estimator. The model that we used is constructed as follows:

(1) $$\begin{array}{c} \Delta Development_{i}=\alpha +\beta Treatment_{i}+\delta Development_{i,baseline}+\gamma X_{i}+\varepsilon_{i} \end{array}$$

where i is an index for a child, $\Delta Development_{i}$ is the change in the cognitive and social–emotional standardized scores of the child i between two survey waves (Wave 1 and Wave 2, Wave 1 and Wave 3, Wave 2 and Wave 3). $Treatment_{i}$ represents treatment. In our analysis, we have two different treatments, as discussed above, namely: strengthened parental beliefs and weakened parental beliefs. $Development_{i,baseline}$ represents the standardized cognitive score or social–emotional score of a child i at baseline wave. The variable $X_{i}$ is a vector of covariates that capture demographic characteristics, including the child’s age (in months) and gender; whether the child was premature (born before 37 weeks of gestation); whether the child had a low birth weight; whether the child’s mother was the primary caregiver; the primary caregiver’s age; the primary caregiver’s education level; and whether the family received social security support.

In addition to the DID estimator, we also adopted a difference-in-difference matching (DIDM) approach to check the robustness of the DID results. The DIDM is an approach extended from the cross-sectional propensity score matching (PSM) approach to a longitudinal setting [43]. The DIDM estimator takes the first differences in the outcomes for the treated and comparison observations and thereby removes any variation in time-invariant unobserved characteristics between treatment and comparison observations [44].

To examine if parenting practices played a mediating role in the impact of parental beliefs on child development; that is, to what extent the impact of strengthened or weakened parental beliefs on child development can be explained by the impact of strengthened or weakened parental beliefs on parenting practices, we followed a standard mediation analysis process [45]. The models are constructed as follows:

(2) $$\begin{array}{c} \Delta Parenting_{i}=\alpha_{0}+\alpha_{1}Treatment_{i}+\gamma X_{i}+\mu_{i} \end{array}$$

(3) $$\begin{array}{c} \Delta Development_{i}=\beta_{0}+\beta_{1}\Delta Parenting_{i}+\beta_{2}Treatment_{i}+\gamma X_{i}+\varepsilon_{i} \end{array}$$

where $\Delta Parenting_{i}$ is the changes in parenting practices scores of the primary caregiver of a child *i* between two survey waves (Wave 1 and Wave 2, Wave 1 and Wave 3, Wave 2 and Wave 3). The terms $X_{i}$ are defined as above in Equation (1). $\alpha_{1}$ is the estimate of strengthened or weakened parental beliefs on the mediator, or the changes in parenting practices. $\beta_{1}$ is the marginal effect of the mediator on the changes in child developmental outcomes. First, we estimated $\alpha_{1}$ by regressing the effect of the treatment on the mediator. We then estimated $\beta_{1}$ from the regression of child developmental outcomes on treatment. Finally, we computed the 95% Monte Carlo confidence intervals for ($\alpha_{1}$ × $\beta_{1}$) the indirect effect. Confidence intervals that do not include zero indicate a significant indirect effect of the mediator on child developmental outcomes.

## 3. Results

### 3.1. Descriptive Statistics of Child Development, Parental Beliefs, and Parenting Practices

Table 3 reports the descriptive statistics of child development, parental beliefs, and parenting practices in three survey waves. As indicated in Table 3, BSID scores were below population norms at both Wave 1 and Wave 2. In addition, BSID scores declined from Wave 1 to Wave 2. When the children were 22–36 months old, their GMDS-ER scores were within the normal limits. When the children were 49–65 months old, their cognitive scores (WIPPSI score) were below the population norm. In the case of child social–emotional development, the ASQ:SE scores increased from Wave 1 to Wave 3. In terms of parenting practices, the scores in three waves were around 1. That is, the caregivers in our sample, on average, only conducted one of four parenting practices.

### 3.2. Impact of Parental Beliefs on Child Developmental Outcomes

Table 4 presents the results of the analysis examining the effect of parental beliefs on child developmental outcomes (both cognitive and social–emotional development) for the three different time periods using the DID approach (from the Wave 1 survey to the Wave 2 survey; from the Wave 1 survey to the Wave 3 survey; and from the Wave 2 survey to the Wave 3 survey). The results suggest that neither strengthened parental beliefs nor weakened parental beliefs significantly affected child cognitive developmental outcomes in any of the three time periods (Columns 1–3). The coefficients of interest that measured the direct effect of parental beliefs on child cognition were all insignificantly different from zero (Rows 1 and 2).

In the case of child social–emotional development, however, the findings were different (Columns 4–6; Rows 1 and 2). The results show the significant impacts of parental beliefs on the social–emotional development of the sample children in all three time periods. Specifically, compared to children raised by primary caregivers who had the parental beliefs scores below the mean score in both the baseline and endline waves, children raised by primary caregivers who experienced strengthened parental beliefs had a significant decrease in social–emotional standardized scores during the time period of the Wave 1 survey to the Wave 3 survey (Column 5; Row 1) and the Wave 2 survey to the Wave 3 survey (Column 6; Row 1). In contrast, compared to children raised by primary caregivers who had parental beliefs scores above the mean score in both baseline and endline waves, children raised by primary caregivers who experienced weakened parental beliefs had a significant increase in social–emotional standardized scores (worsening social–emotional abilities) during all three time periods (Columns 4–6; Row 2). In other words, according to the findings, between the different waves of the survey, strengthened parental beliefs improved child social–emotional development, while weakened parental beliefs deteriorated child social–emotional development.

### 3.3. Mediation Analysis of Impact of Parental Beliefs on Child Developmental Outcomes through Parenting Practices

When carrying out the analysis using the standardized mediation model that was described in the methods section, Table 5 presents the effect sizes of parental beliefs on parenting practices. The findings show that there is a significant and positive impact of strengthened parental beliefs on parenting practices between the time period of the Wave 1 of the survey and the Wave 2 of the survey (Column 1; Row 1). Compared to primary caregivers with parental beliefs scores lower than the mean in both survey waves, primary caregivers with strengthened parental beliefs had higher levels of parenting practices. The effect size was 0.18 and was significant at a 5% level of significance. Similarly, the results also find a significant and negative impact of weakened parental beliefs on parenting practices between the time period of the Wave 1 survey and the Wave 2 survey. Specifically, compared to primary caregivers who had parental beliefs scores higher than the mean in both the Wave 1 survey and the Wave 2 survey, primary caregivers with decreasing parental beliefs scores between the time period of the Wave 1 survey and the Wave 2 survey had significant lower levels of parenting practices. The effect size of the weakened parental beliefs on parenting practices was −0.35, and this was significant at the 1% level.

No significant impact, however, was found in other cases. Specifically, no specific impact was found in the cases of the strengthened parental beliefs or the weakened parental beliefs during the other two sets of time periods (i.e., between the Wave 1 survey and the Wave 3 survey, or between the Wave 2 survey and the Wave 3 survey).

Table 6 provides the results of mediating the effect of parental beliefs on child social–emotional developmental outcomes through parenting practices. In either treatment group (strengthened parental beliefs or weakened parental beliefs), no significant result was found between any of the three sets of time periods.

Table 7 presents the estimates of the indirect effects of parental beliefs on child social–emotional development through parenting practices in the period between the Wave 1 survey and the Wave 2 survey. The results show that, in the case of the strengthened parental beliefs, the point estimate was negative; however, all the 95% CI (Percentile), 95% CI (BC), and the 95% CI (BCa) overlapped with zero. According to these results, there were no indirect effects of strengthened parental beliefs on child social–emotional development through parenting practices. In the case of the weakened parental beliefs, however, the results show that the point estimate was positive, and that all the 95% CI (Percentile), 95% CI (BC), and 95% CI (BCa) did not overlap with zero. In this case, the results suggest that there was a significant indirect effect of weakened parental beliefs on child social–emotional development through parenting practices between the period of the Wave 1 survey and the period of the Wave 2 survey.

### 3.4. Robustness Check with Alternative Estimator

Table 8 provides the results of a robustness check of the impact of parental beliefs on child developmental outcomes using an alternative approach, the DIDM estimator. The findings from the DIDM-based analysis reveal that there was no significant impact of parental beliefs on child cognitive developmental outcomes, either in the case of the strengthened parental beliefs treatment or in the case of the weakened parental beliefs, between any of the three sets of time periods. This finding is consistent with the results we obtained when using the DID estimator (as shown above in Table 4). In contrast, the DIDM analysis did show that there was a significant impact of parental beliefs on child social–emotional developmental outcomes. Specifically, when compared against children whose primary caregivers had parental beliefs scores lower than the mean both in the baseline survey and in the endline survey, children raised by primary caregivers with strengthened parental beliefs had higher levels of social–emotional development in the time period between the Wave 1 survey and the Wave 3 survey, and in the time period between the Wave 2 survey and the Wave 3 survey. Similarly, children raised by primary caregivers with weakened parental beliefs had significantly lower levels of social–emotional development between all three sets of time periods when compared to children whose primary caregivers had parental beliefs scores higher than the mean, both in the baseline survey and in the endline survey. The effect sizes for the three time periods were 0.33 (between the Wave 1 survey and the Wave 2 survey), 0.30 (between the Wave 1 survey and the Wave 3 survey), and 0.23 (between the Wave 2 survey and the Wave 3 survey), respectively. These findings from using the DIDM approach are similar to what was found when using the DID approach.

We also used the DIDM approach to check the robustness of the results of the mediation analysis (Appendix A Table A1 and Table A2). The results show that, between the time period of the Wave 1 survey and the Wave 2 survey, parental beliefs had a significant impact on parenting practices. These findings are also consistent with what was found in the mediation analysis using the DID approach.

## 4. Discussion

Based on longitudinal data covering three different stages from early childhood (18–30 months), middle childhood (22–36 months), and late childhood (49–65 months) in rural China, the overall goal of this study was to understand the importance of parental beliefs in the process of child development. In pursuing this goal, we had four specific objectives. The first objective was to investigate the levels of child development. The second objective was to assess the levels of parental beliefs and parenting practices in rural China. The third objective was to understand if there was a positive (or negative) impact on either a child’s cognitive abilities or social–emotional skills, if parental beliefs in the importance of parental investment in cultivating a supporting environment for children was improving (or deteriorating) between key sets of time periods when children were between 1.5 and 5 years old. The fourth objective was to investigate to what extent the impact of parental beliefs on child development was delivered by parenting practices.

Comparing the changes in the parental beliefs scores of primary caregivers between the three sets of time periods, we found that when parental beliefs strengthened, there was a positive and significant impact on child social–emotional development. In contrast, when parental beliefs weakened, there was a negative and significant impact on child social–emotional development. No significant impacts, however, were found in the case of child cognitive development in either “treatment” (a strengthening set of parental beliefs or a weakening set of parental beliefs). Despite a perception that is commonly found in the literature, our findings show that there was only a marginal impact on child development that can be indirectly explained by parental beliefs through enhanced or intensified parenting practices.

The findings of this study show that, in any of the three survey waves, the levels of child development (in both cognitive skills and social–emotional skills), parental beliefs, and parenting practices were low. In a healthy population the same age as our sample children, the mean scores of cognitive skills are expected to be 100 with a standard deviation of 15. However, the means of the cognitive scores of our sample children were over one deviation lower than the reference mean scores of a healthy population. In addition, the mean scores of the social–emotional development of our sample children at different age ranges were much higher than the cutoffs set by the ASQ:SE manual, which means that our sample children had much lower levels of social–emotional development compared to a healthy population. In the case of parental beliefs, the average score of primary caregivers in our sample was just a little higher than the expected average score. In terms of parenting practices, the average score was less than one, which was far lower than the expected average score.

The findings of our quasi-experimental analysis (DID) suggest that, compared to children whose primary caregivers had low parental beliefs scores over the course of the survey waves (that is, lower than the scores of the sample means in all three survey waves), children whose primary caregivers experienced strengthened parental beliefs scores were shown to have improved social–emotional skills. Similarly, compared to children whose primary caregivers had stable high parental beliefs scores (that is, higher than the mean scores over the course of three survey waves), children whose primary caregivers had weakened parental beliefs scores were shown to have deteriorating social–emotional developmental outcomes. These findings are consistent with previous studies conducted in the U.S and Ethiopia where the research found that parents who reported that they understood the importance of early childhood development, had children with higher levels of measured developmental outcomes [23,24,25,26]. The findings of those studies therefore demonstrated that parental beliefs impacted not only a child’s social–emotional development, but also a child’s cognitive development. In the case of our study, however, we did not find significant impacts of parental beliefs on child cognitive developmental outcomes. This inconsistency with previous studies may lie in the fact that according to the literature conducted in the U.S, child social–emotional development has greater malleability than cognitive development [16,17]. This implies that the improvement in parental beliefs may, over a longer period of time, have an impact on a child’s cognitive development.

Our findings also showed that parental beliefs had significant impacts on parenting practices when the child was at an early stage of childhood. Specifically, when parental beliefs were growing stronger over the course of the study, the results showed that parenting practices improved. In contrast, when parental beliefs weakened between waves of the survey, it had a negative impact on parenting practices. In fact, these findings are consistent with previous studies (that were run in Colombia, the UK, and Chile) that have shown that parents with stronger parental beliefs in the importance of child development (which was associated in some of the studies with parental training interventions) led to improved parenting practices [6,27,28,29]. However, our study only found such an impact in the very early stages of childhood (between Wave 1 and Wave 2 of the survey) instead of across the period that the surveys covered (i.e., between approximately 2 years old and 5 years old). The possible reasons for this finding might be that even though strengthened parental beliefs improved parenting practices, primary caregivers did not interact with their child consistently. This implies that we must deliver more information about child development to parents in rural China and make primary caregivers more aware of the positive activities, such as talking, singing songs, and interacting in specific ways with their child, have on the development of their child. We must also keep them interacting with their child across the course of their childhood.

The findings of the mediation analysis showed that in our sample, parental beliefs affected child development outcomes only in a relatively small way with a mediating role through parenting practices. Interestingly, this finding is inconsistent with previous studies in Colombia and the UK that have shown that parenting practices are an effective channel through which parental beliefs can affect child development [6,28]. The possible reason may be that the overall levels of parenting practices were quite poor in our sample. Hence, it could be that the quantity and the quality of parenting practices by primary caregivers were so poor that even when parental beliefs strengthened, it was not good enough to improve child developmental outcomes significantly.

We acknowledge three limitations of this study. First, although this study selected a large sample and covered a relatively long time period, children and their primary caregivers were all from western rural China. Future research needs to include children and their primary caregivers from other parts of rural China to make the sample more representative. Second, this study measured the frequencies of a series of parenting practices; however, the quality of the parenting practices was not assessed. Future research needs to use other measurement approaches to evaluate both the quantity and the quality of the parenting practices, as well as to investigate the role that the quality of parenting practices can play in a child’s development. Third, since this study is focused on an Asian population, readers should be cautious in using the findings of this study when referring to other populations under the age of 5.

## 5. Conclusions

This study demonstrated that parental beliefs do have significant impacts on child social–emotional developmental outcomes. In addition, parental beliefs do affect (albeit in a relatively small way) child social–emotional skills through parenting practices when children are young. Future studies should examine the effect of interventions that aim at providing more information about the importance of child development and other parental beliefs on child developmental outcomes. Furthermore, future studies should investigate the effect of interventions that aim to improve the quantity and the quality of parenting practices in rural China on child development. By doing so as early as possible, young rural children in China could have higher levels of development at earlier stages of life that could lead them on a more optimal path of future development.

## Figures and Tables

**Table 1:** Comparison of mean characteristics of treatments and comparisons (treatments: strengthened parental beliefs; comparisons: stable low parental beliefs).

Characteristic	Between Wave 1 and Wave 2			Between Wave 1 and Wave 3			Between Wave 2 and Wave 3		
	Treatment	Control	Difference (1)–(2)	Treatment	Control	Difference (4)–(5)	Treatment	Control	Difference (7)–(8)
	Mean	Mean	*p*-Value	Mean	Mean	*p*-Value	Mean	Mean	*p*-Value
	(1)	(2)	(3)	(4)	(5)	(6)	(7)	(8)	(9)
**Child**									
Age in months	23.95	23.44	0.099	23.66	23.66	0.979	29.15	29.16	0.977
Male	0.49	0.53	0.426	0.52	0.51	0.916	0.52	0.51	0.773
Premature	0.06	0.05	0.640	0.05	0.05	0.862	0.05	0.05	0.983
Low birth weight	0.04	0.05	0.740	0.06	0.03	0.202	0.05	0.05	0.983
**Household**									
Primary caregiver	0.65	0.59	0.227	0.70	0.55	0.002	0.56	0.53	0.526
Caregiver age	32.12	32.75	0.563	32.34	32.58	0.832	33.14	32.98	0.879
Caregiver education	0.31	0.38	0.140	0.36	0.34	0.640	0.44	0.36	0.102
Household receives social security	0.25	0.28	0.532	0.24	0.28	0.268	0.24	0.27	0.527
*N*	181	243		174	250		177	263	

**Table 2:** Comparison of mean characteristics of treatments and comparisons (treatments: weakened parental beliefs; comparisons: stable high parental beliefs).

Characteristic	Between Wave 1 and Wave 2			Between Wave 1 and Wave 3			Between Wave 2 and Wave 3		
	Treatment	Control	Difference (1)–(2)	Treatment	Control	Difference (4)–(5)	Treatment	Control	Difference (7)–(8)
	Mean	Mean	*p*-Value	Mean	Mean	*p*-Value	Mean	Mean	*p*-Value
	(1)	(2)	(3)	(4)	(5)	(6)	(7)	(8)	(9)
**Child**									
Age in months	23.88	23.59	0.381	23.76	23.72	0.898	29.30	29.27	0.918
Male	0.49	0.52	0.511	0.49	0.51	0.729	0.50	0.51	0.893
Premature	0.05	0.03	0.449	0.03	0.04	0.551	0.03	0.05	0.480
Low birth weight	0.05	0.05	0.788	0.05	0.05	0.986	0.03	0.06	0.158
**Household**									
Primary caregiver	0.62	0.72	0.041	0.66	0.69	0.503	0.57	0.68	0.026
Caregiver age	32.29	30.39	0.049	31.49	31.21	0.769	32.06	31.43	0.521
Caregiver education	0.40	0.26	0.003	0.35	0.31	0.320	0.33	0.24	0.063
Household receives social security	0.24	0.22	0.604	0.24	0.21	0.522	0.26	0.21	0.234
*N*	197	194		186	205		173	202	

**Table 3:** Child development, parental beliefs, and parenting practices (*N* = 815).

Variable	Wave 1 (18–30 Months) Mean (SD)	Wave 2 (22–36 Months) Mean (SD)	Wave 3 (49–65 Months) Mean (SD)
	(1)	(2)	(3)
**Child developmental outcomes**			
BSID score	85.34 (20.00)	82.02 (21.04)	
GMDS-ER score		105.00 (21.06)	
WIPPSI-IV score			87.75 (11.52)
ASQ:SE score	61.06 (35.25)	73.33 (41.67)	80.33 (44.72)
**Parental beliefs**			
Total parental beliefs score	47.66 (6.34)	47.46 (6.28)	48.59 (6.04)
**Parenting practices**			
Total parenting practices score	0.91 (1.04)	1.10 (1.21)	0.67 (0.97)

Note. The total parental beliefs score is calculated by summing the scores of the 12 items. The total parenting practices score is calculated by summing the scores of the 4 items.

**Table 4:** DID analysis of the effect of strengthened and weakened parental beliefs on child developmental outcomes.

Variable	ΔStandardized Cognitive Score			ΔStandardized Social–Emotional Score		
	Between Wave 1 and Wave 2	Between Wave 1 and Wave 3	Between Wave 2 and Wave 3	Between Wave 1 and Wave 2	Between Wave 1 and Wave 3	Between Wave 2 and Wave 3
	(1)	(2)	(3)	(4)	(5)	(6)
Treatment 1: Strengthened parental beliefs	0.08	0.17	0.11	−0.13	−0.44 ***	−0.49 ***
	(0.11)	(0.09)	(0.08)	(0.10)	(0.09)	(0.09)
*N*	424	424	440	424	424	440
Adj. *R*^2^	0.31	0.40	0.35	0.36	0.41	0.38
Treatment 2: Weakened parental beliefs	−0.15	0.02	−0.14	0.35 ***	0.30 ***	0.22 **
	(0.09)	(0.09)	(0.10)	(0.09)	(0.11)	(0.09)
*N*	391	391	375	391	391	375
Adj. *R*^2^	0.35	0.30	0.35	0.43	0.40	0.41
Baseline child development	Yes	Yes	Yes	Yes	Yes	Yes
Controls	Yes	Yes	Yes	Yes	Yes	Yes
County FE	Yes	Yes	Yes	Yes	Yes	Yes

Note. Standard errors are clustered at village level. ** *p* < 0.05, *** *p* < 0.01.

**Table 5:** Impact of strengthened and weakened parental beliefs on parenting practices.

Variable	ΔParenting Practices Factor z-Score		
	Between Wave 1 and Wave 2	Between Wave 1 and Wave 3	Between Wave 2 and Wave 3
	(1)	(2)	(3)
Treatment 1: Strengthened parental beliefs	0.18 **	0.08	0.13
	(0.09)	(0.10)	(0.09)
*N*	424	424	440
Adj. *R*^2^	0.28	0.32	0.42
Treatment 2: Weakened parental beliefs	−0.35 ***	−0.11	0.02
	(0.11)	(0.11)	(0.11)
*N*	391	391	375
Adj. *R*^2^	0.50	0.44	0.45
Baseline child development	Yes	Yes	Yes
Controls	Yes	Yes	Yes
County FE	Yes	Yes	Yes

Note. Standard errors are clustered at village level. ** *p* < 0.05, *** *p* < 0.01.

**Table 6:** Mediation analysis of impact of parental beliefs on child social–emotional development through parenting practices.

Variable	ΔStandardized Social–Emotional Score		
	Between Wave 1 and Wave 2	Between Wave 1 and Wave 3	Between Wave 2 and Wave 3
	(1)	(2)	(3)
Treatment 1: Strengthened parental beliefs	−0.12	−0.44 ***	−0.50 ***
	(0.10)	(0.09)	(0.09)
ΔParenting practices factor z-score	−0.06	−0.02	0.07
	(0.04)	(0.04)	(0.05)
*N*	424	424	440
Adj. *R*^2^	0.36	0.41	0.38
Treatment 2: Weakened parental beliefs	0.34 ***	0.32 ***	0.22 **
	(0.09)	(0.11)	(0.09)
ΔParenting practices factor z-score	−0.02	0.07	0.00
	(0.03)	(0.04)	(0.04)
*N*	391	391	375
Adj. *R*^2^	0.43	0.40	0.41
Baseline child development	Yes	Yes	Yes
Controls	Yes	Yes	Yes
County FE	Yes	Yes	Yes

Note. Standard errors are clustered at village level. ** *p* < 0.05, *** *p* < 0.01.

**Table 7:** Estimates of indirect effect of strengthened and weakened parental beliefs on child social–emotional development through parenting practices (Wave 1 to Wave 2).

Indirect Effect Variable	Point Estimate	Bootstrap S.E.	95% CI (Percentile)	95% CI (BC)	95% CI (BCa)
	(1)	(2)	(3)	(4)	(5)
Panel A. Strengthened parental beliefs					
Δ Parenting practices factor z-score	−0.013	0.012	(−0.041, 0.003)	(−0.049, 0.001)	(−0.049, 0.001)
Panel B. Weakened parental beliefs					
Δ Parenting practices factor z-score	0.014	0.008	(0.001, 0.034)	(0.002, 0.034)	(0.001, 0.034)

Note. Bootstrap standard errors reported in Column 2 were based on resampling with 1000 replications. The percentile 95% CI in Column 2 uses usual sampling distribution cutoffs without bias correction; the BC 95% CI in Column 4 corrects for a bias in the distribution of bootstrap estimates of standard errors; and the BCa 95% CI in Column 5 corrects for bias and skewness in the distribution of bootstrap estimates of standard errors.

**Table 8:** DIDM analysis of the effect of strengthened and weakened parental beliefs on child developmental outcomes.

Variable	ΔStandardized Cognitive Score			ΔStandardized Social–Emotional Score		
	Between Wave 1 and Wave 2	Between Wave 1 and Wave 3	Between Wave 2 and Wave 3	Between Wave 1 and Wave 2	Between Wave 1 and Wave 3	Between Wave 2 and Wave 3
	(1)	(2)	(3)	(4)	(5)	(6)
Treatment 1: Strengthened parental beliefs	0.08	0.17	0.10	−0.13	−0.45 ***	−0.48 ***
	(0.11)	(0.09)	(0.08)	(0.10)	(0.09)	(0.09)
*N*	422	418	432	422	418	432
Adj. *R*^2^	0.32	0.40	0.35	0.34	0.39	0.37
Treatment 2: Weakened parental beliefs	−0.15	0.03	−0.13	0.33 ***	0.30 ***	0.23 **
	(0.09)	(0.09)	(0.10)	(0.09)	(0.11)	(0.09)
*N*	384	387	368	384	387	368
Adj. *R*^2^	0.34	0.30	0.35	0.46	0.41	0.42
Baseline child development	Yes	Yes	Yes	Yes	Yes	Yes
Controls	Yes	Yes	Yes	Yes	Yes	Yes
County FE	Yes	Yes	Yes	Yes	Yes	Yes

Note. Standard errors are clustered at village level. ** *p* < 0.05, *** *p* < 0.01.

## Data Availability

The original contributions presented in the study are included in the article/Appendix A, further inquiries can be directed to the corresponding author/s.

## Appendix A

**Table A1:** DIDM analysis of the effect of strengthened and weakened parental beliefs on parenting practices.

Variable	ΔParenting Practices Factor z-Score		
	Between Wave 1 and Wave 2	Between Wave 1 and Wave 3	Between Wave 2 and Wave 3
	(1)	(2)	(3)
Treatment 1: Strengthened parental beliefs	0.18 **	0.07	0.12
	(0.09)	(0.10)	(0.09)
*N*	422	418	432
Adj. *R*^2^	0.29	0.32	0.41
Treatment 2: Weakened parental beliefs	−0.34 ***	−0.10	0.03
	(0.11)	(0.11)	(0.11)
*N*	384	387	368
Adj. *R*^2^	0.51	0.43	0.44
Baseline child development	Yes	Yes	Yes
Controls	Yes	Yes	Yes
County FE	Yes	Yes	Yes

Note. Standard errors are clustered at village level. ** *p* < 0.05, *** *p* < 0.01.

**Table A2:** Mediation analysis of child social–emotional development using DIDM approach.

Variable	ΔStandardized Social–Emotional Score		
	Between Wave 1 and Wave 2	Between Wave 1 and Wave 3	Between Wave 2 and Wave 3
	(1)	(2)	(3)
Treatment 1: Strengthened parental beliefs	−0.12	−0.45 ***	−0.49 ***
	(0.10)	(0.09)	(0.09)
ΔParenting practices factor z-score	−0.06	−0.02	0.07
	(0.04)	(0.04)	(0.05)
*N*	422	418	432
Adj. *R*^2^	0.35	0.39	0.38
Treatment 2: Weakened parental beliefs	0.33 ***	0.31 ***	0.23 **
	(0.09)	(0.11)	(0.09)
ΔParenting practices factor z-score	−0.01	0.07	0.00
	(0.03)	(0.04)	(0.04)
*N*	384	387	368
Adj. *R*^2^	0.46	0.41	0.41
Baseline child development	Yes	Yes	Yes
Controls	Yes	Yes	Yes
County FE	Yes	Yes	Yes

Note. Standard errors are clustered at village level. ** *p* < 0.05, *** *p* < 0.01.
